# Corrosion Resistance of Titanium Dental Implant Abutments: Comparative Analysis and Surface Characterization

**DOI:** 10.3390/ma16206624

**Published:** 2023-10-10

**Authors:** Jakub Kowalski, Dorota Rylska, Bartłomiej Januszewicz, Bartlomiej Konieczny, Michal Cichomski, Jukka P. Matinlinna, Mateusz Radwanski, Jerzy Sokolowski, Monika Lukomska-Szymanska

**Affiliations:** 1 Department of General Dentistry, Medical University of Lodz, 92-213 Lodz, Poland; 2 Institute of Materials Science and Engineering, Lodz University of Technology, 1/15 Stefanowskiego St., 90-924 Lodz, Poland; dorota.rylska@p.lodz.pl (D.R.); bartlomiej.januszewicz@p.lodz.pl (B.J.); 3 University Laboratory of Materials Research, Medical University of Lodz, 251 Pomorska St., 92-213 Lodz, Poland; bartlomiej.konieczny@umed.lodz.pl; 4 Department of Material Technology and Chemistry, Faculty of Chemistry, University of Lodz, 163 Pomorska St., 90-236 Lodz, Poland; michal.cichomski@chemia.uni.lodz.pl; 5 Biomaterials Science, Division of Dentistry, The University of Manchester, Manchester M13 9PL, UK; jpmat@hku.hk; 6 Department of Endodontics, Medical University of Lodz, 92-213 Lodz, Poland; mateusz.radwanski@umed.lodz.pl

**Keywords:** dental implant, abutment, corrosion resistance, titanium, SEM, EDS

## Abstract

Metals subjected to the oral environment are prone to corrosion over time and this can be harmful. Metallic restoration components such as dental subgingival implant abutments are exposed to pH changes and different ions while in contact with saliva. The aim of the study was to evaluate the corrosion resistance of titanium dental implant abutments and to compare and contrast the surface characteristics of these alloys before and after corrosion. The corrosion examination (E_corr_, j_corr_, OCP, polarization curve) of two implant abutments (TiDesign EV, Astra Tech, Dentsply, York, PA, USA; Individual Titanium Abutment, Apollo Implants Components, Pabianice, Poland) was performed in 0.9% NaCl and 5% HCl. Moreover, specimens were investigated using SEM-EDS before and after the corrosion test. The value of j_corr_ in NaCl was higher for Astra (34.2 × 10^−8^ ± 2.5 × 10^−8^ A/cm^2^) than for Apollo (8.8 × 10^−8^ ± 2.5 × 10^−8^ A/cm^2^). Whereas, in HCl, the opposite relationship was observed (Astra 2.9 × 10^−4^ ± 0.8 × 10^−4^ A/cm^2^ and Apollo 62.7 × 10^−4^ ± 9.3 × 10^−4^ A/cm^2^). An average reactive anodic current density in NaCl for Astra amounted up to ~0.2 × 10^−5^–1.5 × 10^−5^ A/cm^2^, while for Apollo-up to ~3.3–9.7 × 10^−7^ A/cm^2^. The composition of both alloys after corrosion in NaCl demonstrated some changes: a decrease in the Ti, and Al and an increase in oxygen content. Hence, both alloys after corrosion in HCl demonstrated some minor changes in the elemental composition. Based on the results it can be concluded that: 1. Astra and Apollo abutments revealed good corrosion resistance and a passivation layer on the surface. 2. Apollo abutments exhibited better corrosion resistance in a neutral environment, suggesting that Astra abutments were found to be more resistant to corrosion in an acidic medium.

## 1. Introduction

Dental subgingival implants are increasingly used for the reconstruction of missing teeth. The most frequently applied type of restoration is a crown placed on the abutment which is fixed to implant. Abutments have been manufactured of titanium (Ti) or its alloys, gold alloys, zirconium dioxide (ZrO_2_), surgical stainless steel, polyether ether ketone (PEEK) and some other polymers [1,2,3]. Only Ti corrosion is thermodynamically unfavorable which explains its superiority as a dental implant material.

The most investigated and popular materials for implant abutment are titanium alloys: commercially pure (CpTi) grade I-IV and Ti-6Al-4V (grade V) [4]. Ti-6Al-4V is an α+β-titanium alloy. To stabilize both phases, certain additives are included: α-stabilizers are aluminum and oxygen, and β-stabilizers—vanadium, tantalum, molybdenum, iron, nickel, chromium, and niobium [5,6]. This alloy is composed of 5.50–6.75 wt.% of Al, 3.50–4.50 wt.% of V, up to 0.40 wt.% of Fe and up to 0.20 wt.% of oxygen, and the balance of the alloy is Ti [7].

There are many advantages of this alloy, e.g., the thermal expansion coefficient is similar to the bone, and the alloy possesses good corrosion resistance, low density, and good biocompatibility [8,9,10]. Moreover, it exhibits good mechanical properties, namely hardness (349 HV), ultimate tensile strength (950 MPa), yield tensile strength (880 MPa), modulus of elasticity (113.8 GPa), and thermal conductivity (6.7 W/mK) and fatigue strength (240 MPa) [8,9,11]. Moreover, cases of allergy to Ti have been described very rarely, only 0.6% [12,13]. Titanium uniquely provides very good osseointegration due to the surface topography: the moderate roughness of 1–2 µm enhances osseointegration and high wettability [14,15]. On the other hand, some disadvantages of Ti-alloys should be mentioned, namely darkish color, difficulty in casting and machining, and high melting temperature [16].

Undoubtedly, metals subjected to the oral environment are prone to degradation over time. The occurrence of corrosion depends on the type of alloys, technological processes used for their production, laboratory treatment, and oral conditions such as pH (fluid or food, type of corrosive solution), temperature, wear (at the implant-abutment interface), microorganisms and biofilm around the implant and crown restoration [17,18,19]. Additionally, corrosion of Ti alloys can be diminished by surface modification including plasma spraying, anodizing (increasing roughness of titanium), laser treatment (surface development), acid etching (such as sulphuric acid, hydrofluoric acid, and hydrochloric acid), grit-blasting (titanium dioxide, aluminum oxide, zirconium dioxide, and silicon carbide powders), machining and polishing [3,20]. In general, Ti-6Al-4V is resistant to microbial-induced and pitting corrosion, but it is susceptible to crevice corrosion and environmentally assisted cracking (i.e., hydrogen-induced cracking and stress corrosion cracking) facilitating corrosion [19]. Galvanic corrosion may occur in the oral cavity when restorations are made from two different alloys exhibiting different electrochemical potentials. Saliva, as a good conducting medium, facilitates the ion migration. This is why this reaction at the material/electrolyte interface causes ion release from the metal into saliva or oral tissue [21,22,23]. This phenomenon results in structural and mechanical deterioration of the alloy along with intra- and extraoral clinical manifestations [24,25,26]. The former includes a decrease in strength and fatigue resistance, leading to mechanical and structural failure of implants and components. Whereas, the latter comprises local reactions such as staining of the peri-implant tissues, perioral stomatitis, osteolysis, oral oedema, and systemic reactions, namely fatigue, hair loss, eczematous rashes, or episodes of brain-fog [24,25,26].

Hence, in an oxidizing environment (moist air or aqueous solutions) a TiO_2_ layer of 2–20 nm is instantly formed on the Ti-alloy surface providing protection against corrosion [19,27]. These oxide layers may form crystalline (anatase or rutile of structures of TiO_2_) or a non-crystalline oxide film, that is stable in a neutral environment (i.e., saline solution) [19,28,29]. In neutral electrolyte solution, a more homogenous and thicker oxide layer is formed [19]. It is noteworthy that it has been hypothesized that the protective properties of oxide film depend on the structure of the oxide film (be it anatase or rutile) on the Ti-alloy subjected to a corrosive environment [19,30] or on the thickness of the outermost oxide layer [31].

On the other hand, strong reducing mineral acids, such as HF, HCl, and H_2_SO_4_, cause the dissolution of this protective film and the activation of the alloy’s surface making it susceptible to attacks and damage [32,33]. Moreover, a decrease in pH can diminish the value of open circuit potential (OCP), causing further degradation [34]. Moreover, in highly acidic concentrations of HCl, from 1.4 M to 9.9 M, a Ti hydride film is formed on the alloy surface of Ti [32,33]. This film is equally protective, but it does not form spontaneously like anatase and rutile [32]. However, the destruction of the Ti oxide layer can lead to high susceptibility to Cl^−^ ions resulting in damage to the abutment surface [35] because of the penetration and subsequent destabilization of the protective layer and hindrance of the passivation process [36,37].

Furthermore, metallic restorations, such as dental implants and abutments are exposed to different cations (such as Mg^2+^, Ca^2+^, Na^+^, K^+^) and anions (like SO_4_^2−^, Cl^−^, HCO^3−^, H_2_PO^4−^) in the oral cavity [38]. Moreover, these corrosion-contributing factors can facilitate the release of metal ions from abutments into surrounding tissues [39]. Unfortunately, as a consequence, apoptosis, oxidative damage to the cells, and defective gene expression can be induced [40,41]. In turn, a decrease in osteoblastic cell viability and cytotoxic effects, resulting in bone resorption were observed [21,22,23]. Interestingly, the formation of a passivating TiO_2_ layer may moderate toxicity by limiting the release of Ti, Al, and V ions from the alloy into the tissue cells [42].

Therefore, the assessment of the corrosion resistance of implant abutments is of crucial importance since in the oral cavity the local acidity may even reach pH values as low as 2.5 [25]. The aim of this laboratory study was to evaluate the corrosion resistance of Ti dental implant abutments and to compare the surface characteristics of these alloys before and after corrosion.

## 2. Materials and Methods

### 2.1. Samples Groups

**Figure 1:**
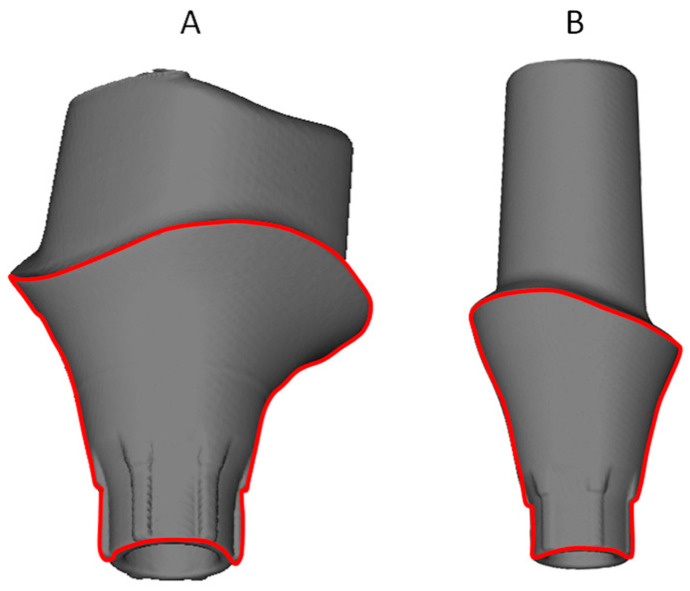
Implant abutment: (**A**)—Apollo, (**B**)—Astra; red line bordering the abutment surface subjected to corrosion examination.

Samples were divided into two groups (*n* = 3): Astra (TiDesign EV, Astra Tech, Dentsply, York, PA, USA) and Apollo (Individual Titanium Abutment, Apollo Implants Components, Pabianice, Poland). Astra and Apollo abutments were made of grade V Ti-alloy (Figure 1). Special tubes (diameter of 14 mm, height of 5 mm) were manufactured using Formlabs Form 2 printer and Formlabs Castable Resin (Formlabs, Somerville, MA, USA). Abutments were introduced into tubes filled with GC Pattern Resin (GC, Tokyo, Japan). The resin-covered cementation reached exactly the border of the transmucosal part. Additionally, the hole in the connection part was filled with resin. The samples were examined under a microscope (25×). The transmucosal and connection parts (surfaces) were left exposed to the corrosion tests and amounted to 80.28 mm^2^ for Astra and 79.83 mm^2^ for Apollo (Figure 1). The surface subjected to corrosion was examined using a micro-CT scanner (Skyscan 1272; Bruker, Kontlich, Belgium) and software NRecon 1.7.4.2 and CTvox 3.3.0 r1403 (Bruker, Kontlich, Belgium).

### 2.2. Corrosion Examination

The study used the following media: 0.9% NaCl (Braun, Melsungen, Germany) and 5% HCl aqueous solution (WarChem, Warsaw, Poland). The tests were performed at room temperature (25 °C) in naturally aerated conditions. The corrosion resistance of the abutment samples was assessed using the Atlas 0531 Electrochemical Unit and Impedance Analyzer (Atlas–Sollich, Rebiechowo, Poland) with the AtlasCorr software 2016, version 2.24 (Atlas-Sollich, Rebiechowo, Poland). An electrochemical cell (CEC/TH Thermostated Multipurpose Cell—Radiometer Analytical, Lyon, France) was used to perform corrosion tests.

Samples were precisely degreased in acetone, then ultrasonically cleansed with deionized water, dried and immediately transferred to a corrosive environment. Next, they were placed in a teflon holder. For the electrochemical measurements, a three-electrode cell with samples (Ti-6Al-4V) as the test electrodes, was applied. A Pt electrode and a silver chloride Ag/AgCl electrode (3M KCl) were used (with a standard potential at 25 °C equal to +0.220 V) as the counter electrode and the reference electrode, respectively. Next, the OCP tests were also carried out for 2 h. The Tafel extrapolation method in the range of ±200 mV vs. OCP was applied to determine corrosion current density (j_corr_) and corrosion potential (E_corr_) in AtlasLab (Atlas-Sollich, Rebiechowo, Poland). Measurements of corrosion parameters were commenced after establishing the OCP (after 120 min from the moment of immersion in corrosive solutions). Potentiodynamic polarization was recorded by scanning from an initial potential of −0.6 V vs. OCP (which was measured against the Ag/AgCl electrode) to the final potential of 1.6 V vs. OCP at a scan rate of 1 mV/s. Electrochemical tests were performed three times for each sample to ensure the reproducibility of the results.

### 2.3. SEM-EDS Study

The metallographic study was performed using a Secondary Electron (SE) detector of JEOL JSM-6610LV scanning electron microscope (JEOL, Peabody, MA, USA), with the accelerating voltage amounting up to 20 keV. The investigation was carried out in several magnifications ranging from 500× up to 2500×.

An EDS X-MAX 80 microanalyzer (Oxford Instruments, Abingdon, UK) was applied to perform the qualitative and quantitative microanalysis of the chemical constitution.

Three samples of each implant abutment study group (Astra and Apollo) were evaluated before and after the corrosion tests.

## 3. Results

### 3.1. Corrosion Resistance

**Figure 2:**
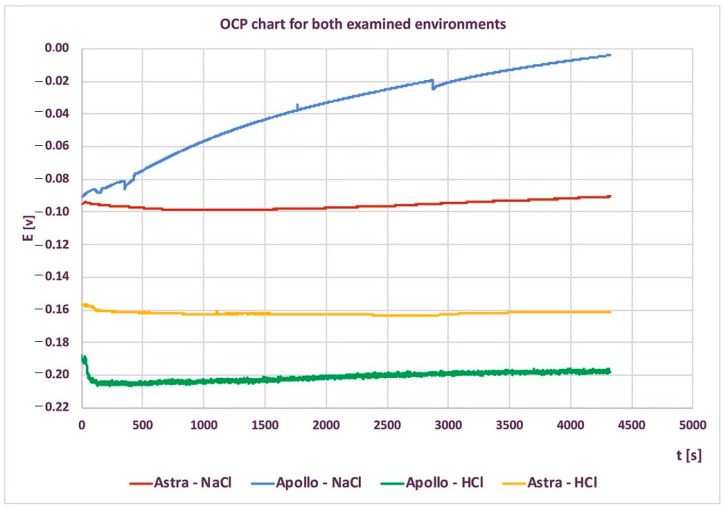
Changes of the stationary potential of both types of abutments as a function of time (solution of 0.9% NaCl and 5% HCl).

The free potential changes as a function of time for study groups are shown in Figure 2. The OCP curves showed an upward trend for the NaCl environment, especially in the case of Apollo. In this medium, the potential value of Apollo (−0.004 V) was higher than Astra (−0.09 V) abutments. Moreover, the growth dynamics for Apollo (+0.086 V) were also significantly higher than for Astra (+0.005 V).

The final values of the OCP in an HCl solution amounted to −0.19 V and −0.157 V for Apollo and Astra abutments, respectively. Shortly after immersion the values slightly increased with time up to −0.16 V for Astra and −0.20 V for Apollo. The final decline in OCP value in HCl was minor and was 0.009 V for Apollo and 0.003 V for Astra abutments.

Table 1 presents corrosion parameters for Astra and Apollo abutments. The values of the current corrosion density and corrosion potential in corrosive environments are estimated using the Tafel extrapolation method. The values of the corrosion potential (E_corr_) in NaCl for Astra and Apollo abutments were equal to −141.6 mV and −174.5 mV, respectively. E_corr_ values determined in HCl were comparable and amounted to −272.6 ± 7.2 mV for Astra and −261.6 ± 3.7 mV–for Apollo.

The corrosion current density (j_corr_) measured in NaCl was higher for Astra (34.2 × 10^−8^ ± 2.5 × 10^−8^ A/cm^2^) than for Apollo (8.8 × 10^−8^ ± 2.5 × 10^−8^ A/cm^2^). Whereas, in HCl, the opposite relationship was observed (Astra 2.9 × 10^−4^ ± 0.8 × 10^−4^ A/cm^2^ and Apollo 62.7 × 10^−4^ ± 9.3 × 10^−4^ A/cm^2^).

**Figure 3:**
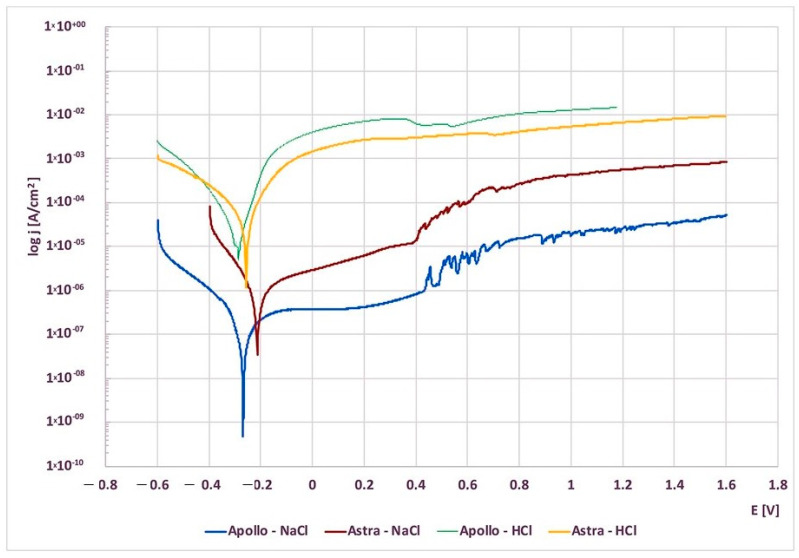
Potentiodynamic polarization curves of Apollo and Astra abutments in NaCl and HCl environments.

The potentiodynamic characteristics for polarization in the range from −0.6 V up to 1.6 V at the potential change rate of 1 mV/s were shown in the semi-logarithmic system (Figure 3). The potentiodynamic curves for Astra (both in NaCl and HCl) were shifted in the positive direction in comparison to Apollo. The anode current of Apollo (~8.5 × 10^−3^) was slightly higher than for Astra (~3.2 × 10^−3^) in HCl, while the opposite relationship was observed in NaCl.

Moreover, the potentiodynamic curves for both study groups in NaCl solution revealed the presence of a well-defined passive region that ranged from −0.2 V up to 0.43 V. An average reactive anodic current density in NaCl for Astra amounted up to ~0.2 × 10^−5^–1.5 × 10^−5^ A/cm^2^, while for Apollo–up to ~3.3–9.7 × 10^−7^ A/cm^2^.

In NaCl above the value of 0.43 V, the current showed the first rapid increase of a relatively small value up to 3.5 × 10^−6^ A/cm^2^ for Apollo and up to 2.5 × 10^−5^ A/cm^2^ for Astra abutments. With further increase in the anode potential in NaCl, slight oscillations of the current density were observed for both study groups.

The HCl polarization curves exhibited also a passive characteristic, which indicated the formation of a passive layer on the surfaces. The anode current densities remained almost unchanged over the wide anodic polarization range; hence significantly higher than in NaCl (Astra ~3.2 × 10^−3^ A/cm^2^, Apollo ~8.5 × 10^−3^ A/cm^2^). There was no rapid increase observed in the anodic current up to the value of the anode potential of 1.6 V in HCl.

### 3.2. SEM-EDS Study

**Figure 4:**
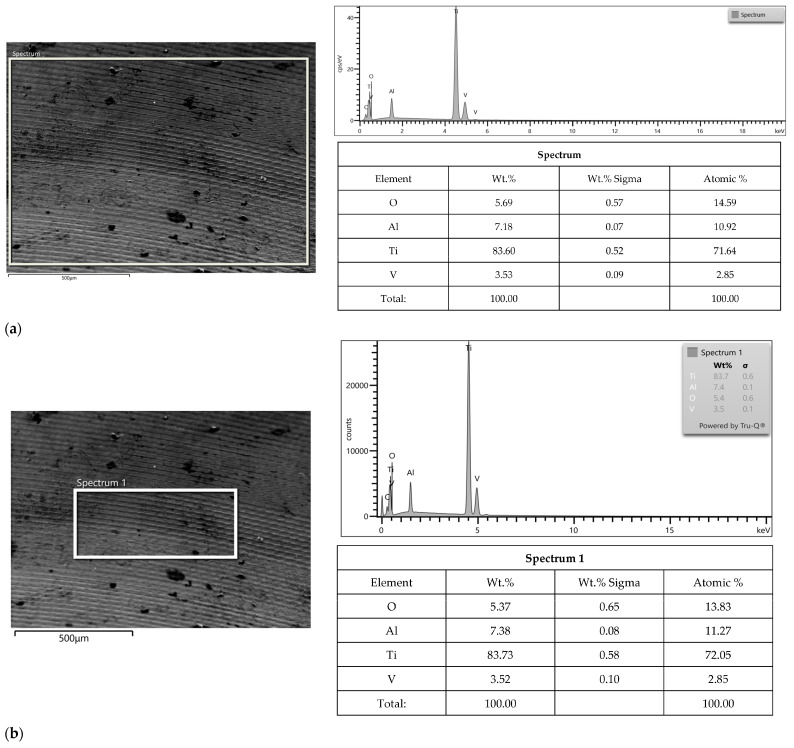

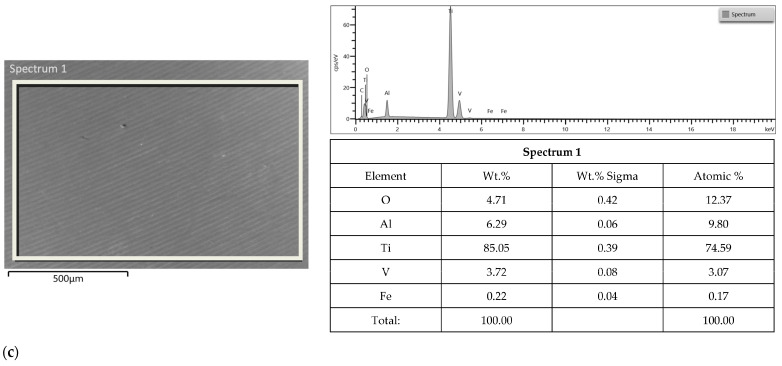
SEM micrographs showing morphology of presented area of Apollo abutment (**a**), an example of smaller area (**b**) and Astra abutments (**c**) before corrosion; EDS results from the presented area before corrosion.

**Figure 5:**
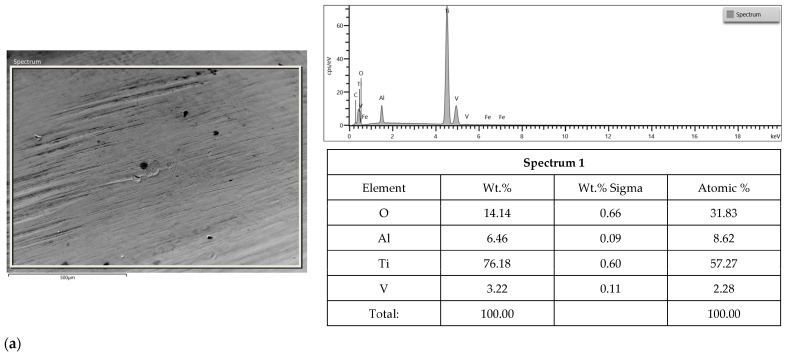

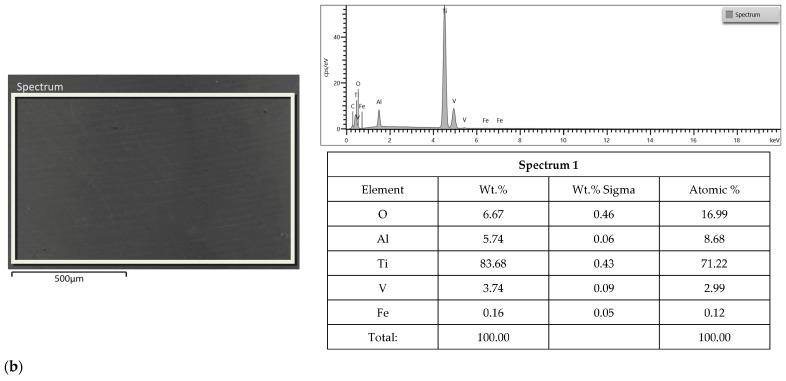
SEM micrographs showing morphology of presented area of Apollo (**a**) and Astra abutments (**b**) after corrosion in NaCl; EDS results for the presented areas are after corrosion.

**Figure 6:**
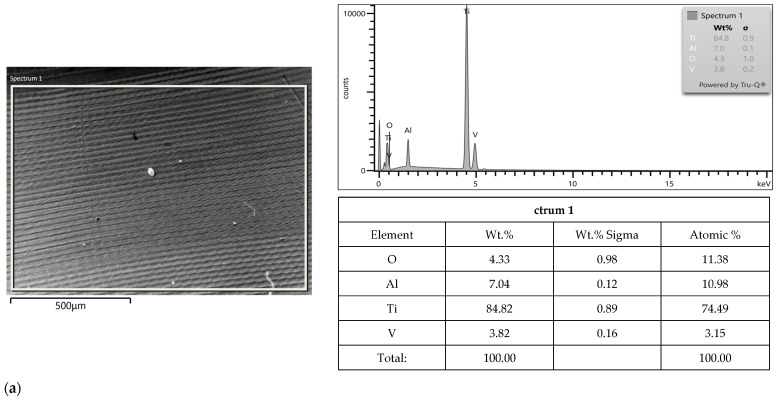

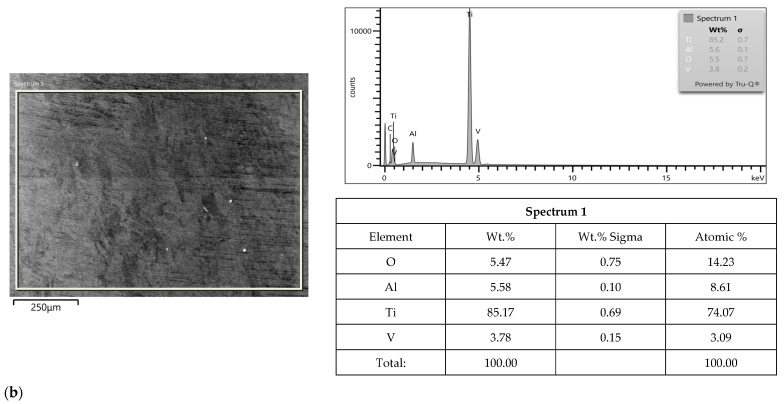
SEM micrograph showing morphology of presented area of Apollo (**a**) and Astra abutments (**b**) after corrosion in HCl; EDS results are from the presented area after corrosion.

Surface morphologies of Apollo and Astra abutments before and after the corrosion test in NaCl and HCl solution are shown in Figure 4, Figure 5 and Figure 6. Before corrosion, the investigated surfaces of Astra and Apollo abutments were compact and uniform with the presence of grooves and ridges (Figure 4). The EDS results revealed element contents (Figure 4). Moreover, the Ti content in Apollo amounted up to 83.6 wt.% and in Astra abutments 85.1 wt.%. Oxygen was present in both alloys (5.7 wt.%—Apollo, 4.7 wt.%—Astra). The percentage of V is comparable in both alloys: a slightly lower value was observed for Apollo (~0.2 wt.%) than for Astra. Hence, the content of Al was by 1.0 wt.% higher in Apollo than in Astra. Moreover, Astra comprised 0.2 wt.% of Fe.

After corrosion in the NaCl solution, the SEM examination revealed grooves for both Apollo (parallel and irregular) and Astra (parallel and perpendicular) (Figure 5a,b). EDS study revealed a typical composition for both alloys. A decrease in the Ti content was observed for Apollo (by 7.4 wt.%) and for Astra (by 1.4 wt.%). Next, a decrease in the Al content was observed for Apollo (by 0.7 wt.%) and for Astra (by 0.6 wt.%). The oxygen content was noted for both alloys, 14.1 wt.% for Apollo and 6.7 wt.% for Astra. Additionally, for Apollo, a slight decrease in the V content and lack of Fe was detected. Whereas the content of the latter remained unchanged in Astra. Altogether, the composition of both alloys after corrosion in NaCl demonstrated some changes: a decrease in the Ti, and Al and an increase in the oxygen content. In general, Apollo exhibited greater changes in the element content than Astra.

After corrosion in the HCl solution, SEM images revealed a few small and parallel grooves for Apollo and irregular grooves for Astra (Figure 6a,b). A slight decrease in Al (by ca. 0.1 wt.% for Apollo and ca. 0.7 wt.% for Astra) was observed. The oxygen content was noted for both alloys −4.3 wt.% for Apollo and 5.5 wt.% for Astra. Additionally, Fe was not observed in Astra. Altogether, the composition of both alloys after corrosion in HCl demonstrated some minor changes, hence smaller than in NaCl. Moreover, Apollo exhibited greater changes in elemental content after corrosion in HCl than Astra.

## 4. Discussion

In the current laboratory study, both investigated alloys revealed good corrosion resistance. This finding is of great clinical importance because ion release is expected to be negligible in the case of these components. As a result, the alloy does not structurally and mechanically deteriorate providing consistent strength and fatigue resistance during clinical service. Moreover, the implant and abutment would be surrounded by healthy soft and hard tissues with no signs of inflammation or lesions. Moreover, the average corrosion potentials for both study groups were similar in the same environment with lower values in HCl than in NaCl. This finding can suggest similar corrosion resistance for both alloys. As suspected, corrosion resistance was higher in NaCl than in HCl. This finding is vastly supported by the literature [28,32,34,43,44,45].

Firstly, Astra (with the alloy with a lower Al content and a minor Fe content) exhibited lower corrosion current density (suggesting more favorable corrosive resistance) in HCl than Apollo (with the alloy with a higher Al content). The possible explanation for these results can be the difference in the Al contents between both alloys. Al (a known α-stabilizer) was proven to play a detrimental role in passivity and the passive film formation of α-Ti in a strong reducing acid (5M HCl) [46]. Moreover, according to the literature, both Al influence the potential range at which hydrides and in particular TiH_2_ are formed in HCl [47]. The addition of Al shortens the range of its existence but, unfortunately, activates its formation. Therefore, the efficiency of the passivating layer is reduced [47]. Interestingly, it was suggested that the detrimental effect of Al (inducing corrosion) in HCl might dominate over other factors [48].

In the present study, the content of V was comparable in both abutments. Hence, other authors reported that this element (a known β-stabilizer) increased the dissolution (in H_2_SO_4_) of the oxide layer due to the defects and ionic conductivity, which increased when the alloying elements (i.e., V) diffused into the oxide layer [19,48]. Consequently, the corrosion of the alloy increased. As a result of the oxidization process, small amounts of V_2_O_5_, VO_2_ and V_2_O_3_ at the outermost surface were formed [49,50]. These V oxides exhibited inferior protective properties than Ti oxides resulting in deterioration of the alloy passivity in a highly acidic environment (i.e., HCl) [5,49,51]. On the other hand, V was proved to impede TiH_2_ formation in HCl resulting in the enhanced efficiency of the passivating layer [47].

Moreover, α+β alloys (grade V Ti alloys) are the most susceptible to corrosion in HCl due to the high solubility and high diffusion of hydrogen in the β-phase [19]. Consequently, the rate of corrosion depends on the volume fraction of the β-phase and the morphology of the microstructure in these alloys [19,52,53,54,55]. It supports the abovementioned hypothesis that V, being a β-stabilizer, decreases the corrosion resistance. Interestingly, studies have presented inconclusive results on whether the α-phase [5,50] or the β-phase [48] had a more detrimental effect on corrosion resistance in HCl. One study suggested that due to the harmful effect of Al (inducing corrosion), the α-phase is preferentially dissolved in HCl relative to the β-phase of Ti_6_Al_4_V [48]. Hence, it has been argued that the β-phase was preferentially dissolved from the base metal (due to the higher V content) in HCl when compared to the α-phase [56]. Moreover, the different rates of film formation are observed on the α- and β-phases. Consequently, in HCl the film is fractured at α/β interfaces [32,57] making these locations the origin of pit development on grade V Ti-alloys [5,56].

In the present study, Apollo abutments (an alloy with a higher Al content) exhibited a lower corrosion current density suggesting more favorable corrosion resistance than Astra (an alloy with lower Al contents and a minor Fe content) in NaCl. However, the literature reporting the corrosion of alloys containing Al is inconclusive [5,49,58,59]. On the one hand, oxides of Al formed on Ti alloys exposed to NaCl dissolved and were believed to be responsible for the increased corrosion susceptibility of these alloys [58]. It is worth mentioning that despite the insignificant difference in the V content, this element (being mandatory to stabilize the β-phase) [5,49] was proved to be detrimental to the passivity and corrosion resistance of the Ti–6Al–4V in Hank’s solution.

Finally, a higher corrosion current density was observed for Astra abutments in NaCl than for Apollo abutments, while the reverse relationship was noted in HCl. It is worth mentioning that the same correlation between alloys and environments was found for the current density in the anodic range. These findings suggest a more favorable corrosive resistance of Astra abutments in HCl and Apollo implant abutments in NaCl.

The shift of OCPs (in both study groups) in the negative direction in an HCl environment demonstrated the dissolution of the native oxide film [5,33]. The highest decrease of OCP was noted for Apollo in HCl, which may imply surface activation associated with the start of the dissolution/thinning process of the outermost passive layer. Also, a small reduction in OCP was observed for Astra abutments in HCl. Literature confirms such behavior of Ti-alloys in the environment with reducing acids [56,60]. Hence, after ca. 1000–2000 s the passive layer started regaining its protective properties to some extent. A similar slight decrease in the OCP value for Astra abutments was found in the initial stage of the research in NaCl. With the passage of time, the OCP values increased, which can be interpreted as a progressive increase in corrosion resistance over time.

On the contrary, the OCP of Apollo in NaCl dramatically increased. The OCP value of Apollo in NaCl was higher than Astra (~−0.007 V vs. ~−0.09 V, respectively). The opposite relationship was observed in the HCl environment, where OCP values were significantly lower than in NaCl.

The analysis of potentiodynamic curves indicates that with a further increase in the anode potential slight oscillations of the current density were observed for both groups in NaCl. This phenomenon is associated with the subsequent formation and repassivation of microcavities. The anode current densities in HCl remained almost unchanged over the wide anodic polarization range; hence significantly higher than in NaCl (Astra ~3.2 × 10^−3^ A/cm^2^, Apollo ~8.5 × 10^−3^ A/cm^2^). This difference is expected given the vigorous nature of HCl. The constant level of the anode current densities indicated that the corrosion rate remained almost unchanged. The HCl polarization curves exhibited also passive characteristics, which indicated the formation of a passive layer on the surfaces. Studies investigating Ti grade V in NaCl showed similar corrosion behavior [5,37,61,62,63,64]. Moreover, research on the corrosion behavior of Ti grade V in HCl confirmed the outcomes of this study [5,33,51]. The differences in the results were due to either a higher concentration of the solution [33,48,65] or the application of de-aerated HCl [56]. The abovementioned studies showed an active-passive range proceeding to the passive state on the anode polarization curves. The present research also evaluated the surface characteristics and elemental composition of alloys before and after the corrosion tests. Before corrosion, the investigated surfaces of Astra and Apollo abutments were compact and uniform with the presence of grooves and ridges due to production machining. Additionally, the composition was similar. Hence, the difference was observed in the Al content that was higher (by ~1 wt.%) in Apollo than in Astra abutments. Interestingly, the Al amount in Astra abutments was within the norm for grade V Ti-alloys, whereas, in Apollo, the content was slightly higher (7.2 wt.% vs. 5.50–6.75 wt.%) [7]. Moreover, Astra abutments comprised 0.2 wt.% of Fe, while EDS analysis of Apollo did not show this element. These differences in the compositions probably have influenced the corrosion resistance results.

After corrosion small, infrequent pits and grooves on the surface were observed that might be related to current power oscillations, and are visible on the potentiodynamic curves. These phenomena occur because solutions containing chlorides favor the anodic dissolution of the passive layer [34]. Cl^−^ ions can migrate through the passivating oxide layer and reach the metal-coating interface [37,45,66,67] subsequently forming titanium oxychloride. This build-up of a metal chloride might cause irreversible damage to the oxide coating and subsequently lead to pitting [62]. The ability to reproduce metastable growing pits depends on the amount of chlorides accumulated at the nucleation site [67]. These imperfections may further trigger the corrosion process. Despite the abovementioned mechanism, in the present study, both investigated alloys exhibited no signs of pitting after corrosion tests in NaCl and HCl.

Altogether, the composition of both alloys after corrosion in NaCl demonstrated some changes: a decrease in the Ti, and Al and an increase in the O content. Interestingly, Jáquez-Muñoz et al. [37] reported the lack of oxygen, but some content of Na and Cl on the surface of grade V Ti-alloy after the corrosion test in NaCl solution indicating that both ions were diffused into the alloy surface. Nevertheless, after corrosion in HCl, the composition of both alloys showed some minor changes, hence smaller than in NaCl. Moreover, Astra abutments did not exhibit Fe in the surface composition which is probably due to the penetration to the electrolyte. Interestingly, Apollo abutments exhibited greater changes in the elemental content than Astra.

The present study investigated two abutments made of grade V Ti-alloy: one original and another customized component to verify their corrosion resistance because it has not been studied until now. The potential range was chosen based on the literature [5,43,51,68]. Moreover, two corrosive environments (NaCl and HCl) were applied to mimic natural and simulated extreme conditions (such as acid reflux, and vomiting); 0.9% NaCl was applied in this study referring to a typical physiological fluid. However, its composition does not reflect the complexity of the existing body fluids with ions such as Cl^−^, PO_4_^3−^, CO_3_^2−^, and SO_4_^2−^. On the other hand, a 5% HCl solution allowed us to investigate alloys in a highly acidic environment giving a wider perspective on the performance of these alloys in severe clinical settings.

Some limitations of the present study should, however, be acknowledged. The study evaluated only two Ti-alloys in two corrosive environments. Therefore, a wider range of abutments and media should be examined in the near future. Furthermore, the sample size could be increased, and other shapes of abutments should be included and investigated. Complementary studies in media at different temperatures and in differentiated concentrations and pH measurements should be performed. In addition, other analysis methods could be implemented, e.g., the electrochemical impedance spectroscopy (EIS) allowing a more accurate evaluation of the characteristics and resistance of the passive layer. It might also provide a more exhaustive investigation including the analysis of the influence of porosity on the performance of alloys in the simulated oral cavity environment. Moreover, research on the surface profile could be implemented to provide a deeper insight into the alloy surface.

## 5. Conclusions

The present study suggests and concludes that:

1. Astra and Apollo implant abutments revealed good corrosion resistance and a passivation layer on the surface.
2. Apollo abutments exhibited better corrosion resistance in a neutral environment, hence Astra abutments were found to be more resistant to corrosion in an acidic medium.

## Figures and Tables

**Table 1:** Corrosion parameters.

Apollo Abutments						Astra Abutments					
j_corr_ [A/cm^2^]	E_corr_ [mV]	βa [V/dec]	βc [V/dec]	Rp [Ohm/cm^2^]	CR [mm/yr]	j_corr_ [A/cm^2^]	E_corr_ [mV]	βa [V/dec]	βc [V/dec]	Rp [Ohm/cm^2^]	CR [mm/yr]
NaCl											
8.8 × 10^−8^ ± 2.5 × 10^−8^	−174.5 ± 2.6	788.7 × 10^−3^	102.1 × 10^−3^	44.6 × 10^3^	7.59 × 10^−4^	34.2 × 10^−8^ ± 2.5 × 10^−8^	−141.6 ± 14.0	542.3 × 10^−3^	108.4 × 10^−3^	11.5 × 10^3^	2.95 × 10^−3^
HCl											
62.7 × 10^−4^ ± 9.3 × 10^−4^	−261.6 ± 3.7	421.3 × 10^−3^	155.1 × 10^−3^	7.85	54.10	2.9 × 10^−4^ ± 0.8 × 10^−4^	−272.6 ± 7.2	430.1 × 10^−3^	136.6 × 10^−3^	1.55 × 10^2^	2.50

## Data Availability

The data presented in this study are available on request from the corresponding author.
